# Multi-band terahertz anisotropic metamaterial absorber composed of graphene-based split square ring resonator array featuring two gaps and a connecting bar

**DOI:** 10.1038/s41598-024-58142-3

**Published:** 2024-03-29

**Authors:** Somayyeh Asgari, Tapio Fabritius

**Affiliations:** Optoelectronics and Measurement Techniques Research Unit, Faculty of Information Technology and Electrical Engineering, University of Oulu, Oulu, Finland

**Keywords:** Applied optics, Optical materials and structures, Electrical and electronic engineering

## Abstract

A multi-band anisotropic metamaterial absorber operating in the terahertz (THz) range is constructed using a graphene-based split square ring resonator array featuring two gaps and a connecting bar. The design is meticulously simulated through the finite element method (FEM) using CST Software. Subsequently, an equivalent circuit model (ECM) is introduced, leveraging impedance and transmission lines, and implemented with a rapid MATLAB code to evaluate the absorber’s behavior in the THz spectrum. The proposed absorber, dynamically adjustable through a one-layered resonator array, exhibits a strong linear dichroism response of 99% within a frequency range of 0.3–4 THz. The metamaterial has an absorption rate of 81% for one absorption band in transverse magnetic mode and its three absorption bands in transverse electric mode have an average of 99.3% in each absorption band with absorption over 99%. This absorber holds potential applications in polarization-sensitive devices and THz systems. The ECM model was established to provide an efficient analytical tool for assessing the absorber’s performance, and the FEM simulation results align well with those derived from the ECM.

## Introduction

Anisotropic metamaterials exhibit non-equal responses to the incident waves along some different directions. Metamaterials fall into the category of anisotropic metamaterials if they possess at least one of the following features: anisotropic geometries, anisotropic material within the metamaterial structure, or applying an external magnetic field to the metamaterial. Metamaterials featuring anisotropic geometries do not coincide with their mirror image at some rotational angles^1^.

Graphene, which is a two-dimensional layer derived from graphite, possesses exceptional qualities that render it a favorable option for optical devices and systems. Recently, there have been proposals, analyses, and investigations into anisotropic metamaterials based on graphene. These metamaterials demonstrate tunable anisotropic responses like linear dichroism (LD)^2–5^. Tunable graphene-based terahertz (THz) anisotropic metamaterials can find potential applications in polarization-sensitive devices and systems.

Through the adjustment of the graphene-based metamaterial absorber’s spectral response by the alternation of the applied bias voltage dynamically, graphene-based multi-band absorbers can be employed in a variety of applications. They can be used to reduce electromagnetic interference for next-generation (6G) wireless communication multi-channel systems by absorbing undesired electromagnetic radiation^6^. Moreover, they have the potential to play a crucial role in advancing telecommunication multi-channel systems in the future. In addition to multi-channel telecommunication systems^7^ these multi-band metamaterial absorbers can be used in highly sensitive and selective sensing and imaging, spectroscopy applications^8–13^, filtering^10^, switching^12^, modulation^13^, energy absorbing and energy converting^14^, power detecting^15^, power receiving^16^, and more. However, most of the earlier reported structures have limited number of absorption peaks thus hindering their usability in some applications.

Creating multi-channel systems involves constructing components with multiple absorption bands. Metamaterials commonly achieve this by integrating either multiple layers of resonators or super unit cells to encompass a multi-band spectrum of absorption bands^17–19^. Graphene-based anisotropic metamaterials reported in^2,5^ are dual-band THz absorbers. The absorber in^2^ has two layers of resonators each layer containing one resonator and the absorber in^5^ has two resonators in one layer of resonator. To increase the number of absorption peaks without compromising with simplicity of the structure, the aim of this work was to design a multi-band THz graphene-based anisotropic metamaterial absorber containing simple geometry with few resonator layers and few resonators per unit cell.

There is one paper reporting four THz graphene-based multi-band metamaterial absorbers composed of square split ring resonator arrays with different number of gaps^20^. The absorbers were designed in 0.1–5.5 THz with limited maximum absorption and limited maximum LD response. In this paper, we have designed and developed a multi-band THz graphene-based anisotropic metamaterial absorber which is composed of a square split ring resonator array featuring two gaps and a connecting bar, with increased absorption, LD, and number of absorption bands. In addition, the ECM in this work differs from that reported in^20^.

## Materials and methods

**Figure 1:**
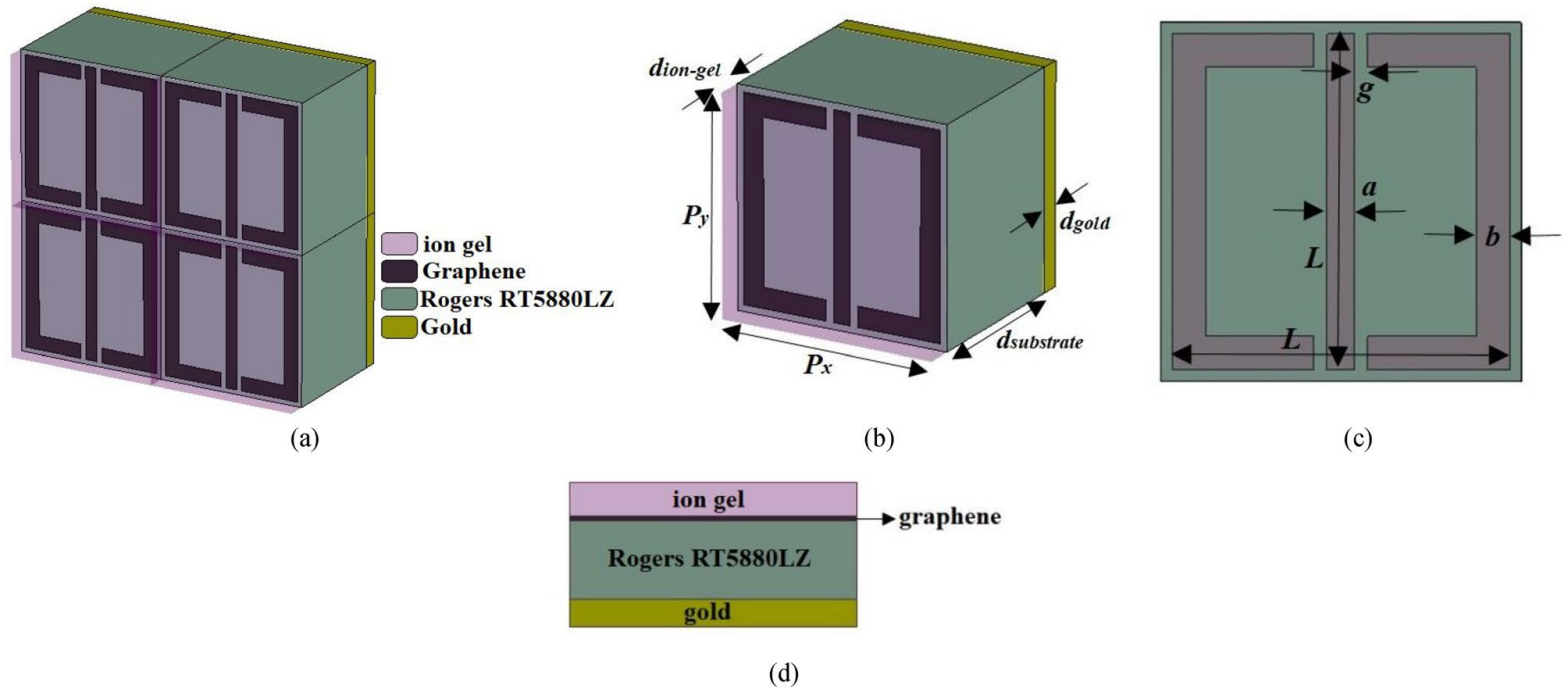
(**a**) Periodic, (**b**) unit cell, (**c**) front, and (**d**) side views of the proposed multi-band terahertz anisotropic metamaterial absorber which is comprised of a graphene-based split square ring resonator array with two gaps and a bar between the gaps. The layers making the metamaterial from the top to the bottom are respectively ion gel, graphene, Rogers RT5880LZ, and gold. We have used a layer of ion gel to bias the resonator array and a gold layer beneath the metamaterial to avoid the transmission of electromagnetic waves.

Figure 1 illustrates the metamaterial absorber configuration from various perspectives, including periodic, unit cell, front, and side views. The layers making the metamaterial from the top to the bottom are respectively ion gel, graphene, Rogers RT5880LZ, and gold. Atop the metamaterial structure, there is a layer of ion gel with a permittivity value of *ε*_*ig*_ = 2.0164 and a thickness of 150 nm. The primary function of this ion gel layer is to apply bias to the underlying graphene resonator layer^21^.

The metamaterial is composed of a gold layer with high conductivity (*σ* = 4.56 × 10^7^ S/m)^22^. The maximum penetration depth ($\delta_{\max } = \sqrt {\frac{1}{{\sigma \pi f_{\min } \mu_{r} \mu_{0} }}}$) for the gold layer happens in 0.3 THz (minimum frequency *f*_*min*_ of the frequency range) where the relative permeability *μ*_*r*_ is 1 and vacuum permeability *μ*_0_ is 4π × 10^–7^. So, the maximum penetration depth is 0.1361 μm. The gold layer with a thickness of 0.5 μm which is much larger than the penetration depth (⁓ 3.7 × maximum penetration depth) of the THz waves is selected to ensure that the incident electromagnetic wave cannot transmit to the other side of the absorber and the transmission is zero because the electromagnetic waves can penetrate inside the gold layer up to its penetration depth^23^. The gold layer acts as a perfect reflector for the considered THz design range. Moreover, it doesn’t produce any loss since it has a big conductivity and energy cannot go through it^24^. Positioned on this gold layer is a dielectric layer, on which graphene patterns are arranged. In this configuration, graphene is depicted as an extremely thin layer (Δ = 0.335 nm) embedded within Rogers RT5880LZ. Rogers RT5880LZ has a permittivity value of *ɛ*_*d*_ = 2. The design also incorporates a graphene-based split square ring resonator array, featuring two gaps and a connecting bar, situated on the dielectric layer.

Fabrication of the metamaterial is not in the scope of this work, but we briefly summarize the fabrication process here for future readers of the work who may want to fabricate this metamaterial: Begin by applying a 13 µm thick dielectric with gold coating on one side through chemical vapor deposition (CVD)^25^. Subsequently, use a standard lithography process to create the graphene patterns on the dielectric^26^. Transfer the ion gel dielectric onto the graphene resonator array using thermal evaporation^27^.

Dimensional and material parameters of the metamaterial of Fig. 1 are optimized. *d*_ion-gel_ is the thickness of the ion gel layer = 150 nm, *d*_substrate_ is the thickness of the dielectric substrate = 13 μm, *d*_gold_ is the thickness of the gold layer = 0.5 μm, *P*_*x*_ is the unit cell dimension in x direction = 16 μm, *P*_*y*_ is the unit cell dimension in y direction = 16 μm, *a* is the width of connecting bar = 1.2 μm, *L* is the length of connecting bar and outer length of split square ring = 15 μm, *g* is the gap distance = 0.6 μm, *b* is the width of split square ring = 1.5 μm, *E*_*f*_ is the Fermi energy level of graphene = 1 eV, and *τ* is the relaxation time of graphene = 2 ps.

Numerical simulations were conducted using the finite element method (FEM) with the frequency domain solver in CST Microwave Studio^5,20,28–30^. Periodic (unit cell) boundary conditions were applied in the x and y directions, while an absorbing (open (add space)) boundary condition was implemented in the z direction. The metamaterial was meshed using a tetrahedral mesh^5,20,29,30^. Optimization of the metamaterial was performed using the genetic algorithm (the parameter values were randomly changed to find the optimal values) within the CST software^5,20,30–32^. The unit cell dimensions were set as P_x_ = P_y_ = 16 μm, a size smaller than *λ*_min_ = 75 μm, which corresponds to *f*_max_ = 4 THz, the upper limit of the simulated frequency range. This sizing decision was made to prevent the propagation of higher-order Floquet modes^5,20,30,33,34^.

The total thickness of the metamaterial, including the ion gel, graphene, Rogers RT5880LZ, and gold layers, was 13.65 μm, approximately 0.182 × *λ*_min_ which shows that the metamaterial is relatively thin in the simulated frequency range^5,30^. The relative permittivity and the surface conductivity of graphene are modeled as reported in^5,20^.

**Figure 2:**
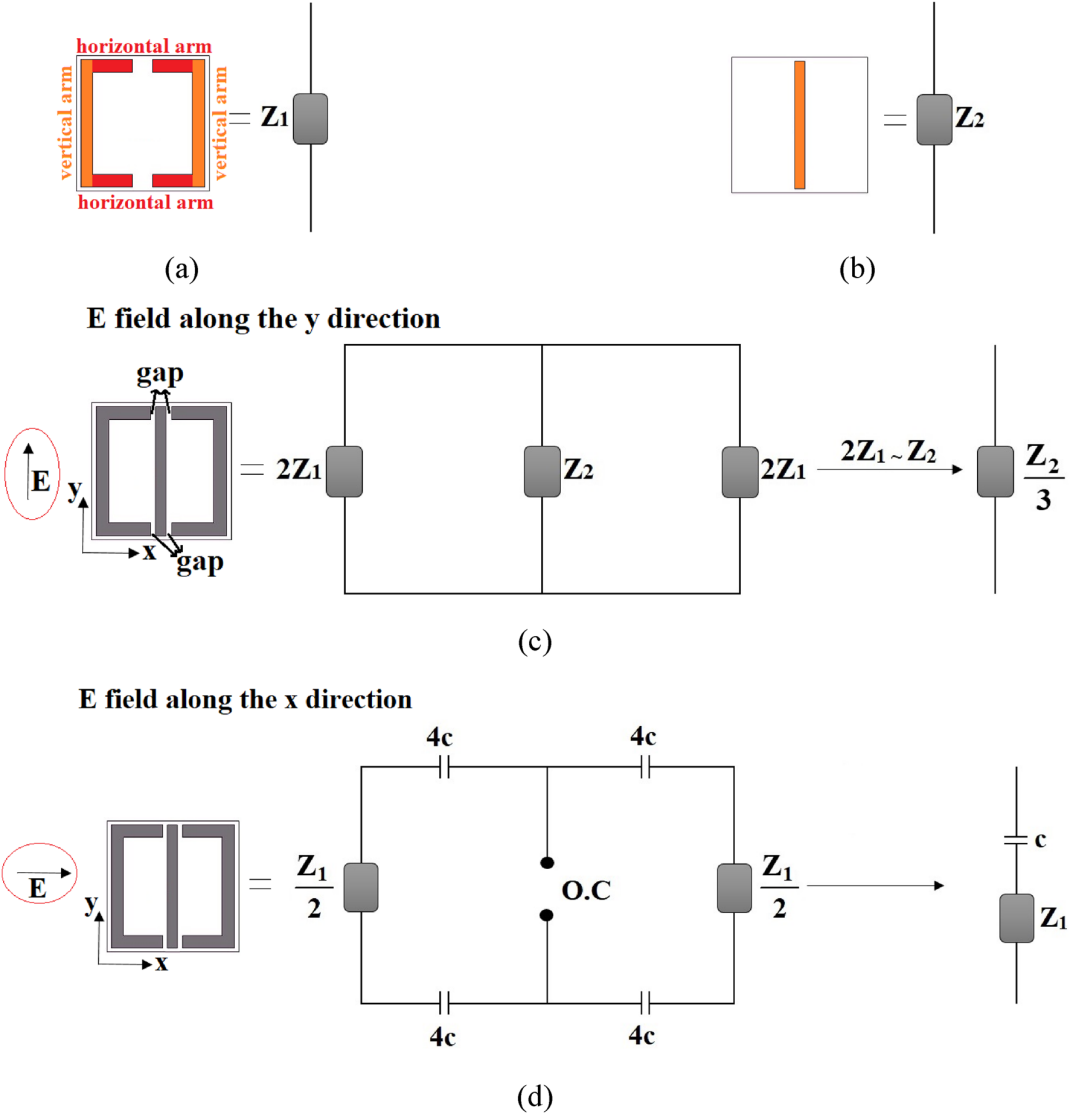
Equivalent circuit model (ECM) of the (**a**) graphene-based split ring resonator array composing of horizontal and vertical arms, (**b**) graphene-based middle connecting bar array, and graphene-based resonator array layer in (**c**) TE (E field along the y direction) and (**d**) TM (E field along the x direction) modes.

As is clear in Fig. 1, the graphene-based resonator array is composed of the graphene-based split ring resonator array and the graphene-based middle connecting bar array. As shown in Fig. 2a,b, the impedance of the split ring resonator array is considered as *Z*_1,_ and the impedance of the middle connecting bar array is assumed as *Z*_2_. The split ring resonator array consists of horizontal and vertical arms as shown in Fig. 2a. The ECM of the graphene-based resonator array in TE and TM modes are respectively given in Fig. 2c,d.

The graphene resonator layer has a non-symmetric geometry so the ECMs in TE and TM modes differ. For TE mode (Fig. 2c), the incident E field is along the y direction. There are four gaps between the middle connecting bar and the split ring resonator (shown in Fig. 2c). The E field is parallel to the gaps, so the gaps are not modeled. E field is along the middle connecting bar, so it’s modeled by *Z*_2_. The split ring resonator is modeled by *Z*_1_ and each half of the split ring resonator is modeled by 2*Z*_1_. As depicted in Fig. 2c, the anisotropic metamaterial contains three parallel impedances in TE mode. So, the impedance in TE mode *Z*_*TE*_ is calculated by:

1 $$Z_{TE} = \frac{{Z_{1} Z_{2} }}{{Z_{1} + Z_{2} }}$$

Since the E field is along the y direction, the produced impedance by the horizontal arms (shown in Fig. 2a) of the split ring resonator is very small and neglected (the width of the arms is much smaller than their length). Each vertical arm (shown in Fig. 2a) of the split ring resonator produces 2*Z*_1_ and they have equal length and width with the middle connecting bar, so they have equal impedances:

2 $$2Z_{1} \sim Z_{2}$$

So:

3 $$Z_{TE} = \frac{{Z_{2} }}{3}$$

For TM mode (Fig. 2d), the incident E field is along the x direction. The E field is normal to the gaps, so the gaps are modeled by capacitors and each capacitor is assumed to have a capacitance of 4c. E field is not along the middle connecting bar, so it’s modeled as an open circuit (O.C). The split ring resonator is modeled by *Z*_1_ and each half of the split ring resonator is modeled by *Z*_1_/2. The ECM in TM mode simplifies to a Seri of *Z*_1_ and c.

The effective conductivity of the graphene resonator layer by Fresnel equation in TE mode can be obtained by^35^:

4 $$\sigma_{TE} = \frac{{\cos \left( {\theta_{{{\text{in}}}} } \right) - \sqrt {\varepsilon_{d} } \cos \left( {\theta_{{{\text{out}}}} } \right) - r^{TE} \left( {\cos \left( {\theta_{{{\text{in}}}} } \right) + \sqrt {\varepsilon_{d} } \cos \left( {\theta_{{{\text{out}}}} } \right)} \right)}}{{Z_{0} \left( {1 + r^{TE} } \right)}}$$

in which *θ*_in_, *ε*_*d*_, *θ*_out_, *r*^*TE*^, and *Z*_0_ are respectively the angle of the incident illuminated wave, the relative permittivity of the dielectric substrate, angle of the transmitted wave, the reflection coefficient of the graphene resonator layer in TE mode, and the vacuum impedance. The relation between *θ*_in_ and *θ*_out_ is:

5 $$\sin \left( {\theta_{{{\text{out}}}} } \right) = \sqrt {\frac{1}{{\varepsilon_{d} }}} \sin \left( {\theta_{{{\text{in}}}} } \right)$$

Impedance is the inverse of conductivity (admittance). So^20^:

6 $$Z_{TE} = \frac{1}{{\sigma_{TE} }}$$

By substituting Eq. (6) in Eq. (3), we have:

7 $$Z_{2} = \frac{3}{{\sigma_{TE} }}$$

which is equal to:

8 $$Z_{2} = \frac{{3Z_{0} \left( {1 + r^{TE} } \right)}}{{\cos \left( {\theta_{in} } \right) - \sqrt {\varepsilon_{d} } \cos \left( {\theta_{out} } \right) - r^{TE} \left( {\cos \left( {\theta_{in} } \right) + \sqrt {\varepsilon_{d} } \cos \left( {\theta_{out} } \right)} \right)}}$$

As shown in Fig. 2b, the graphene resonator array in TM mode is modeled by a series circuit containing Z_1_ and c. So:

9 $$Z_{TM} = Z_{c} + Z_{1}$$

where *Z*_*c*_ is the impedance of the capacitor. Also^20^:

10 $$Z_{TM} = \frac{1}{{\sigma_{TM} }}$$

The effective conductivity of the graphene resonator layer by Fresnel equation in TM mode can be obtained by^35^:

11 $$\sigma_{TM} = \frac{{\sec \left( {\theta_{{{\text{in}}}} } \right) - \sqrt {\varepsilon_{d} } \sec \left( {\theta_{{{\text{out}}}} } \right) - r^{TM} \left( {\sec \left( {\theta_{{{\text{in}}}} } \right) + \sqrt {\varepsilon_{d} } \sec \left( {\theta_{{{\text{out}}}} } \right)} \right)}}{{Z_{0} \left( {1 + r^{TM} } \right)}}$$

where *r™* is the reflection coefficient of the graphene resonator layer in TM mode. The impedance of the gap capacitance can be obtained by^20^:

12 $$Z_{c} = \frac{1}{j\omega c}$$

where *ω* is the angular frequency. The gap capacitance is calculated by:

13 $$c = \varepsilon_{{{\text{eff}}}} \frac{b}{g}$$

where *ε*_eff_ is the effective permittivity calculated by^20^:

14 $$\varepsilon_{{{\text{eff}}}} = \varepsilon_{0} \frac{{1 + \varepsilon_{d} }}{2}$$

in which *ε*_0_ is the permittivity of vacuum. Substituting Eqs. (13) and (14) in Eq. (12), we will have:

15 $$Z_{c} = \frac{2g}{{j\omega b\varepsilon_{0} \left( {1 + \varepsilon_{d} } \right)}}$$

Then, *Z*_1_ can be calculated from:

16 $$Z_{1} = \frac{{Z_{0} (1 + r^{TM} )j\omega l\varepsilon_{0} (1 + \varepsilon_{d} ) - 2w\left[ {\sec \left( {\theta_{{{\text{in}}}} } \right) - \sqrt {\varepsilon_{d} } \sec \left( {\theta_{{{\text{out}}}} } \right) - r^{TM} \left( {\sec \left( {\theta_{{{\text{in}}}} } \right) + \sqrt {\varepsilon_{d} } \sec \left( {\theta_{{{\text{out}}}} } \right)} \right)} \right]}}{{j\omega l\varepsilon_{0} \left( {1 + \varepsilon_{d} } \right)\left[ {\sec \left( {\theta_{{{\text{in}}}} } \right) - \sqrt {\varepsilon_{d} } \sec \left( {\theta_{{{\text{out}}}} } \right) - r^{TM} \left( {\sec \left( {\theta_{{{\text{in}}}} } \right) + \sqrt {\varepsilon_{d} } \sec \left( {\theta_{{{\text{out}}}} } \right)} \right)} \right]}}$$

**Figure 3:**
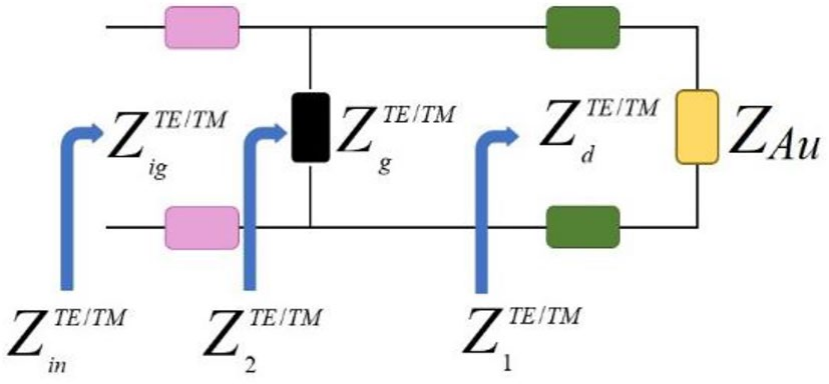
ECM of the whole metamaterial absorber.

The entire metamaterial is represented by transmission lines, as depicted in Fig. 3. The input impedances for each segment of the metamaterial absorber are illustrated in the same figure. Given that the thickness of the graphene layer is significantly smaller than the minimum wavelength within the simulated wavelength range, the graphene resonator layer is simplified and modeled as a point load^36,37^.

The input impedances of the different segments of Fig. 3 are calculated by^20,36^:

17 $$Z_{1}^{TE/TM} = Z_{d}^{TE/TM} \frac{{Z_{Au} + jZ_{d}^{TE/TM} \tan \left( {\beta_{d} d} \right)}}{{Z_{d}^{TE/TM} + jZ_{Au} \tan \left( {\beta_{d} d} \right)}}$$

in which $Z_{d}^{TE/TM}$ and *β*_*d*_ are respectively the TE/TM impedances of the dielectric substrate and the propagation constant of the THz electromagnetic wave in the dielectric substrate.

Since the conductivity of gold is high and its thickness is much larger than the maximum penetration depth of electromagnetic waves in the designed frequency range, the waves cannot transmit through the gold layer to the other side of the metamaterial. So, it acts as a perfect reflector and its impedance can be considered as zero, *Z*_*Au*_ = 0. Then, Eq. (17) is simplified to^20,36^:

18 $$Z_{1}^{TE/TM} = jZ_{d}^{TE/TM} \tan \left( {\beta_{d} d} \right)$$

19 $$Z_{2}^{TE/TM} = Z_{g}^{TE/TM} \left\| {Z_{1}^{TE/TM} } \right.$$

where $Z_{g}^{TE/TM}$ is the TE/TM impedances of the graphene resonator layer. The input impedance of the metamaterial can be obtained by^20,36^:

20 $$Z_{in}^{TE/TM} = Z_{ig}^{TE/TM} \frac{{Z_{2}^{TE/TM} + jZ_{ig}^{TE/TM} \tan \left( {\beta_{ig} d_{ig} } \right)}}{{Z_{ig}^{TE/TM} + jZ_{2}^{TE/TM} \tan \left( {\beta_{ig} d_{ig} } \right)}}$$

in which $Z_{ig}^{TE/TM}$ and *β*_*ig*_ are respectively the TE/TM impedances of the ion gel layer and the propagation constant of the THz electromagnetic wave in the ion gel^20^.

21 $$Z_{ig/d}^{TE} = \frac{{Z_{0} }}{{\sqrt {\varepsilon_{ig/d} } }}\cos \left( {\theta_{in/d} } \right)$$

22 $$Z_{ig/d}^{TM} = \frac{{Z_{0} }}{{\sqrt {\varepsilon_{ig/d} } }}\sec \left( {\theta_{in/d} } \right)$$

in which *θ*_*d*_ is the electrical length of the substrate^20^.

23 $$\theta_{d} = \frac{{d_{{{\text{substrate}}}} \omega \sqrt {\varepsilon_{d} } }}{c}$$

24 $$\beta_{ig/d} = \frac{{\omega \sqrt {\varepsilon_{ig/d} } }}{c}$$

The TE/TM scattering parameters (return losses) of the metamaterial $S_{11}^{TE/TM}$ can be obtained by^20^:

25 $$S_{11}^{TE} = \frac{{Z_{{{\text{in}}}}^{TE} - Z_{0} \cos (\theta_{{{\text{in}}}} )}}{{Z_{{{\text{in}}}}^{TE} + Z_{0} \cos (\theta_{{{\text{in}}}} )}}$$

26 $$S_{11}^{TM} = \frac{{Z_{{{\text{in}}}}^{TM} - Z_{0} \sec (\theta_{{{\text{in}}}} )}}{{Z_{{{\text{in}}}}^{TM} + Z_{0} \sec (\theta_{{{\text{in}}}} )}}$$

in which $Z_{in}^{TE/TM}$ is obtained from Eq. (20). The TE/TM reflections of the metamaterial *R*^*TE/*^*™* are obtained by^20,38^:

27 $$R^{TE/TM} = \left| {S_{11}^{TE/TM} } \right|^{2}$$

Since the metamaterial is backed by a gold layer, there is zero transmission (*T*^*TE/*^*™* = 0) for the metamaterial. So, the insertion losses (*T*^*TE/*^*™* or $S_{21}^{TE/TM}$) for TE/TM modes are zero^38^. The TE/TM absorptions of the metamaterial *A*^*TE/*^*™* are obtained by^20,38^:

28 $$A^{TE/TM} = 1 - R^{TE/TM}$$

The linear dichroism (LD) is the difference in absorbance for TE and TM polarized waves^5,20,38^:

29 $$LD = A^{TM} - A^{TE}$$

## Results and discussion

**Figure 4:**
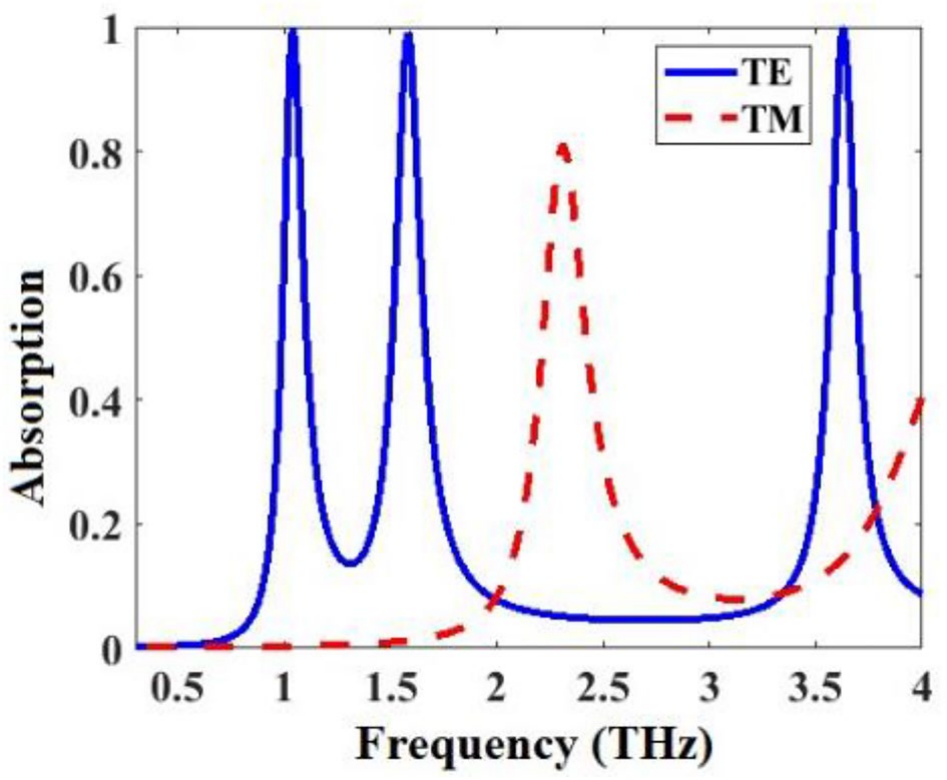
TE and TM absorption spectra of the anisotropic metamaterial absorber of Fig. 1.

Absorption spectra of the anisotropic metamaterial absorber of Fig. 1 for both TE and TM mode are obtained by CST and the results are shown in Fig. 4. The metamaterial has different responses for TE and TM waves, and it works in 0.3–4 THz. There are three resonances in TE mode with average absorption of 99.3% and one resonance in TM mode with absorption rate of 81%.

**Figure 5:**
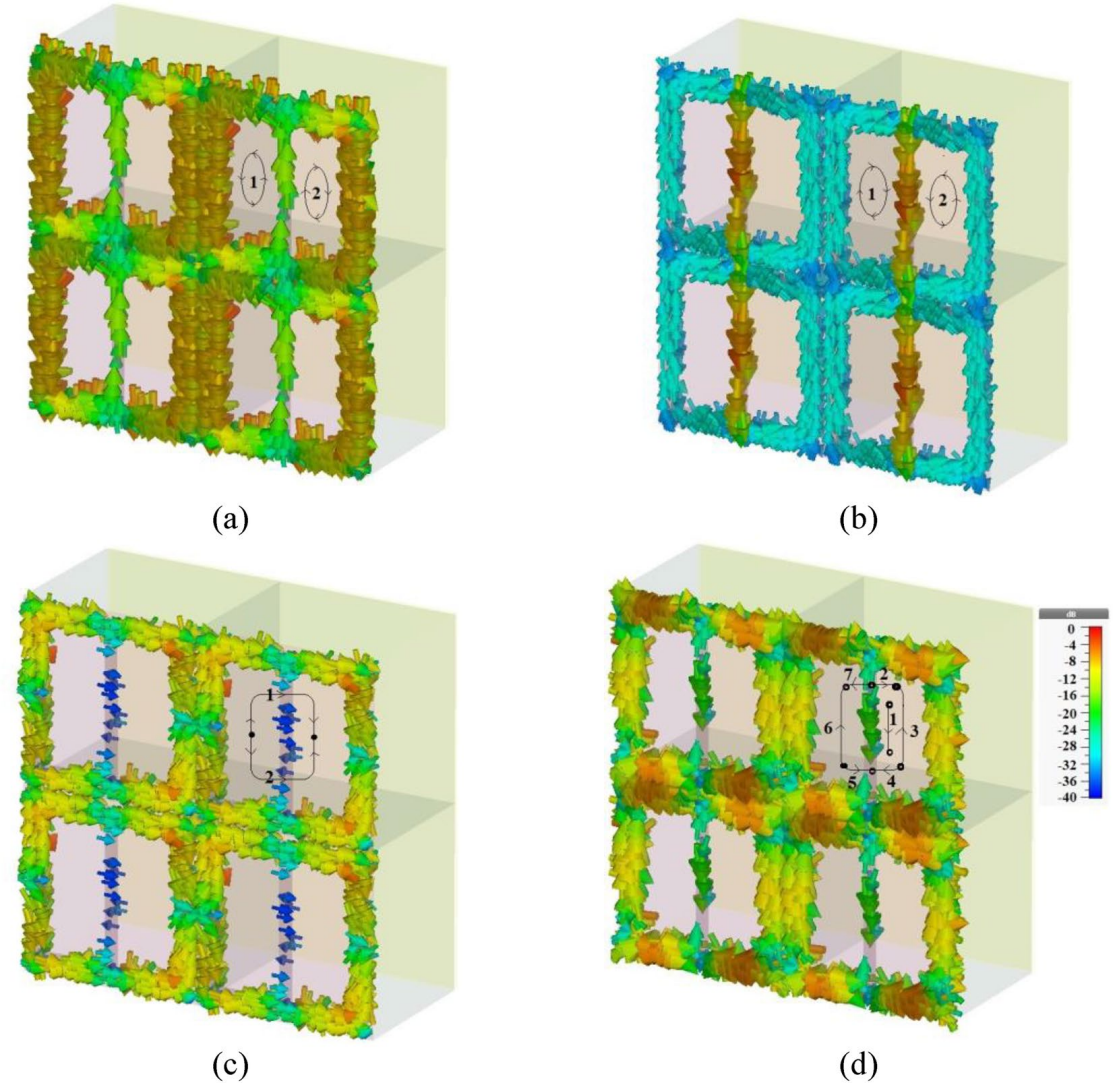
Surface current distributions of the metamaterial absorber of Fig. 1 in (**a**) 1.04, (**b**) 1.58, (**c**) 2.31, and (**d**) 3.63 THz.

Surface current distributions in the resonance frequencies of the anisotropic metamaterial absorber of Fig. 1 are displayed in Fig. 5. In 1.04 THz, the surface currents make two loops (two magnetic dipoles), one of them anticlockwise and the other one clockwise. In 1.58 THz, the surface currents also make two loops (two magnetic dipoles), one of them clockwise and the other one anticlockwise. In 2.31 THz, the surface currents make two electric dipoles with the directions shown in Fig. 5c. In 3.63 THz, the surface currents make seven electric dipoles with the directions shown in Fig. 5d.

**Figure 6:**
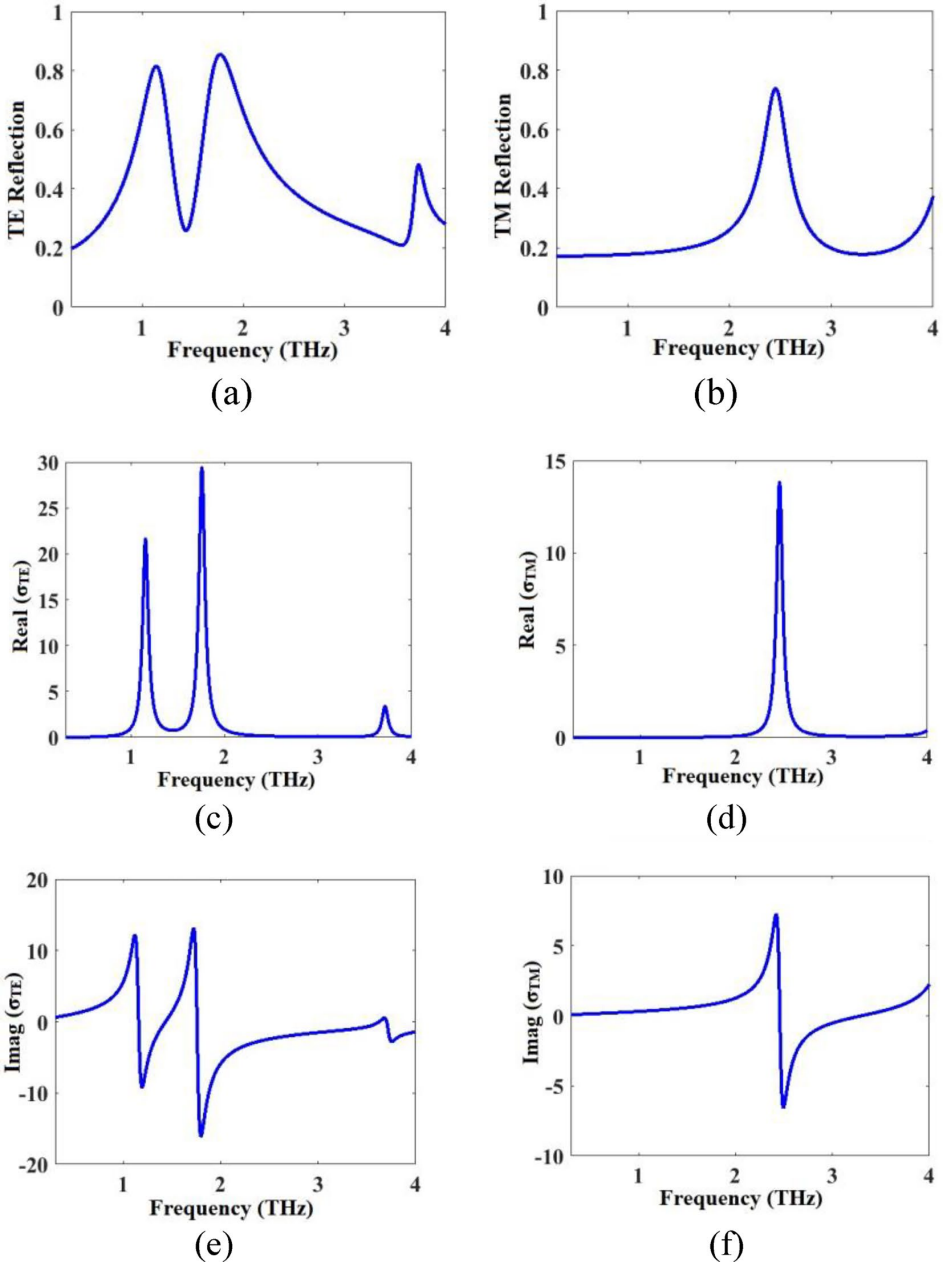
(**a**) TE and (**b**) TM reflection spectra of the graphene-based resonator array layer when its located on the half-space substrate without considering the ion gel and gold layers. (**c**) and (**d**) real, (**e**) and (**f**) imaginary parts of the equivalent conductivity of the graphene resonator array layer in TE and TM modes (Eqs. (10) and (11)).

To start the ECM analysis, the graphene resonator layer is placed on the dielectric with thickness of 300 μm which is considered as a half-space to minimize the influence of the substrate on the impedances of the graphene resonator layer. The configuration is simulated in CST and the reflection spectra are obtained. The obtained TE and TM reflection spectra (*r*^*TE*^ in Eq. (4) and *r™* in Eq. (11)) are given in Fig. 6a,b. Then, Eqs. (4) and (11) are calculated to obtain the equivalent conductivities of the graphene resonator layer in TE and TM modes, and the results are given in Fig. 6c–f. The real parts are positive (Fig. 6c,d) representing the resistive nature and the imaginary parts contain both positive and negative sections (Fig. 6e,f) indicating the inductive and capacitive natures of the graphene resonator array.

**Figure 7:**
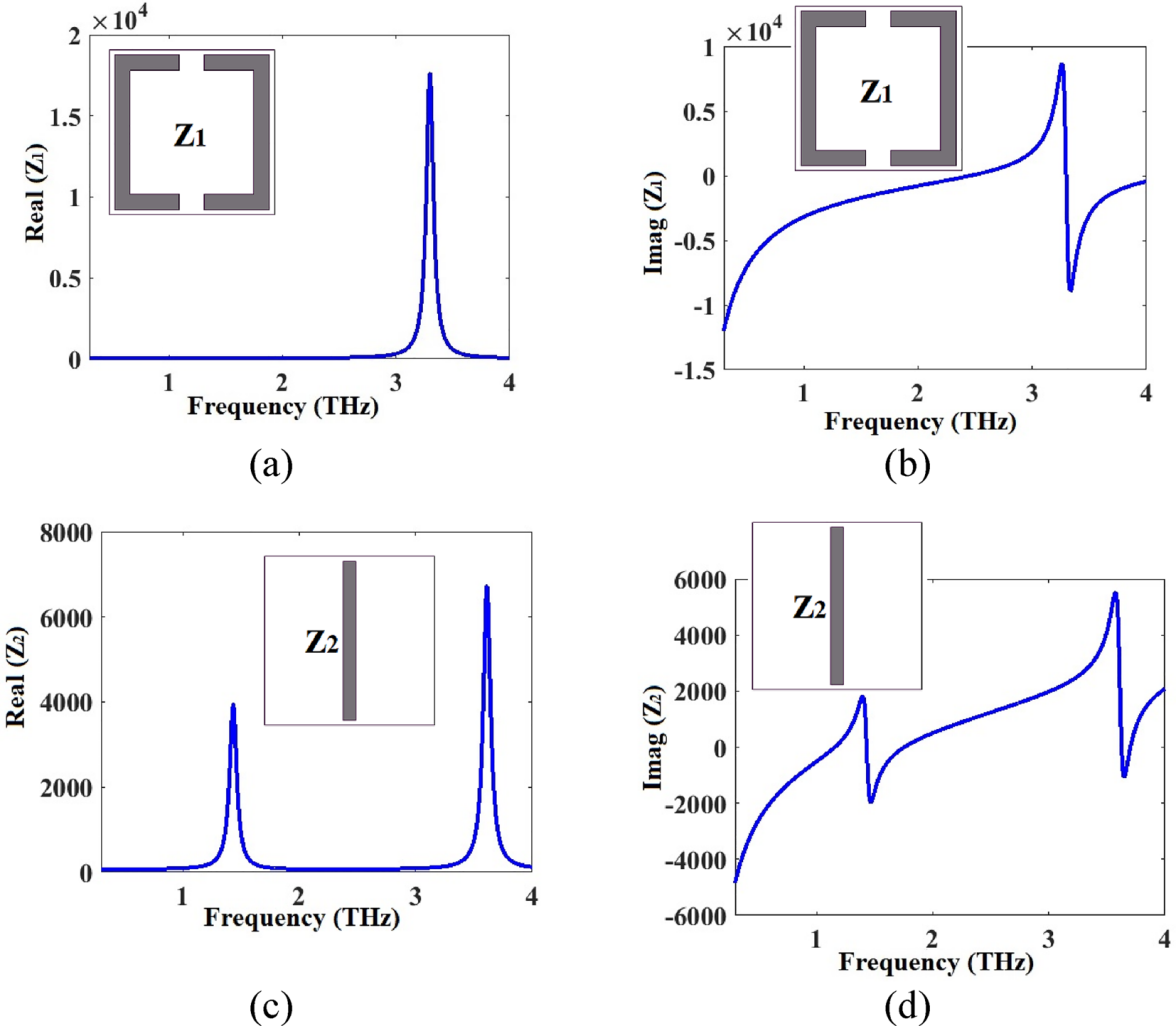
(**a**) Real and (**b**) imaginary parts of Z_1_ (impedance of the split ring resonator array of the metamaterial of Fig. 1 as shown in the inset figure). (**c**) Real and (**d**) imaginary parts of Z_2_ (impedance of the middle connecting bar array of the metamaterial of Fig. 1 as shown in the inset figure).

The resonator array of the metamaterial of Fig. 1 consists of a split ring resonator array *Z*_1_ and the middle connecting bar array *Z*_2_. We have calculated and plotted the real and imaginary parts of the impedances of these arrays separately and the results are given in Fig. 7. *Z*_1_ and *Z*_2_ are shown in the inset figures in Fig. 7. The real parts are positive producing great loss, and the imaginary parts contain both positive and negative sections indicating the inductive and capacitive natures of the split ring resonator array and the middle connecting bar array.

**Figure 8:**
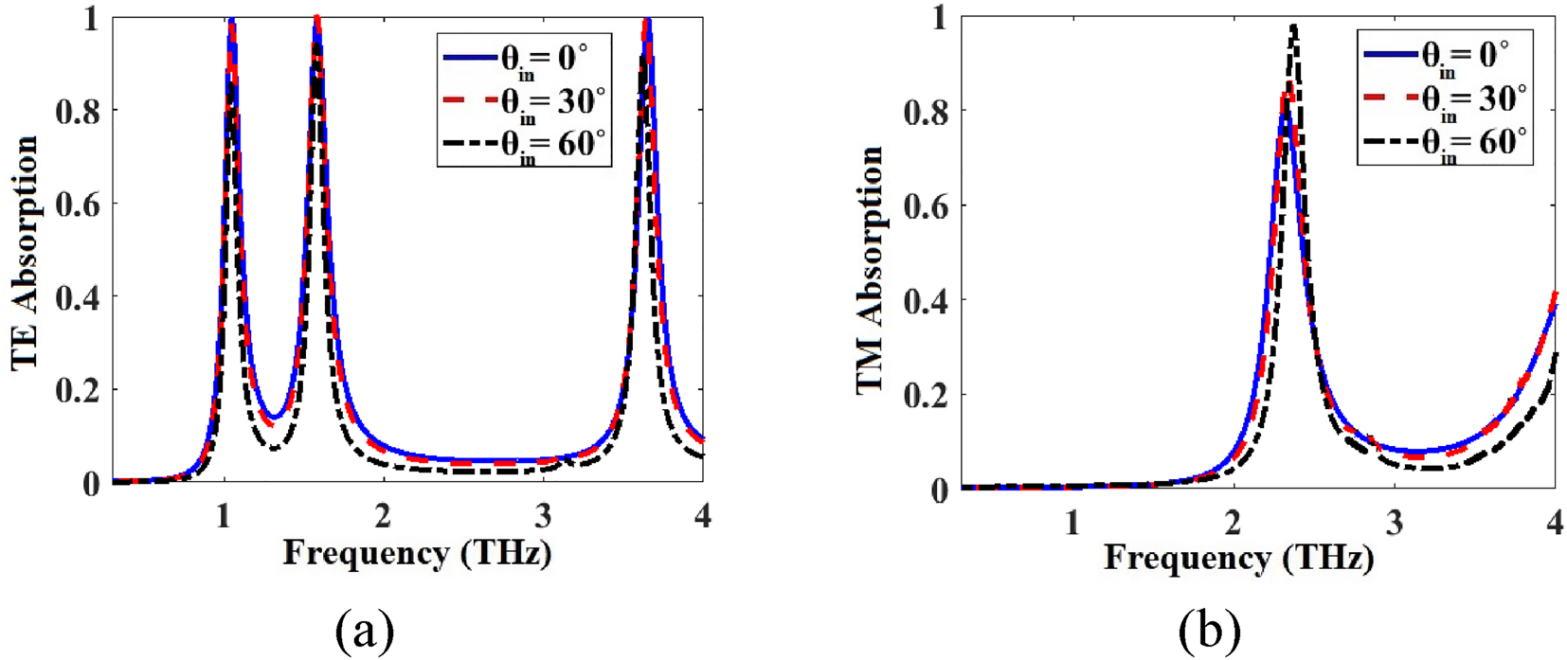
(**a**) TE and (**b**) TM absorption spectra of the metamaterial absorber of Fig. 1 for three different values of incident angle θ_in_.

TE and TM absorption spectra of the metamaterial absorber of Fig. 1 for three different values of incident angle θ_in_ are respectively given in Fig. 8a,b. By change of θ_in_, As shown, by change of θ_in_, the absorption spectra for both modes alter slightly so the metamaterial is incident angle independent.

**Figure 9:**
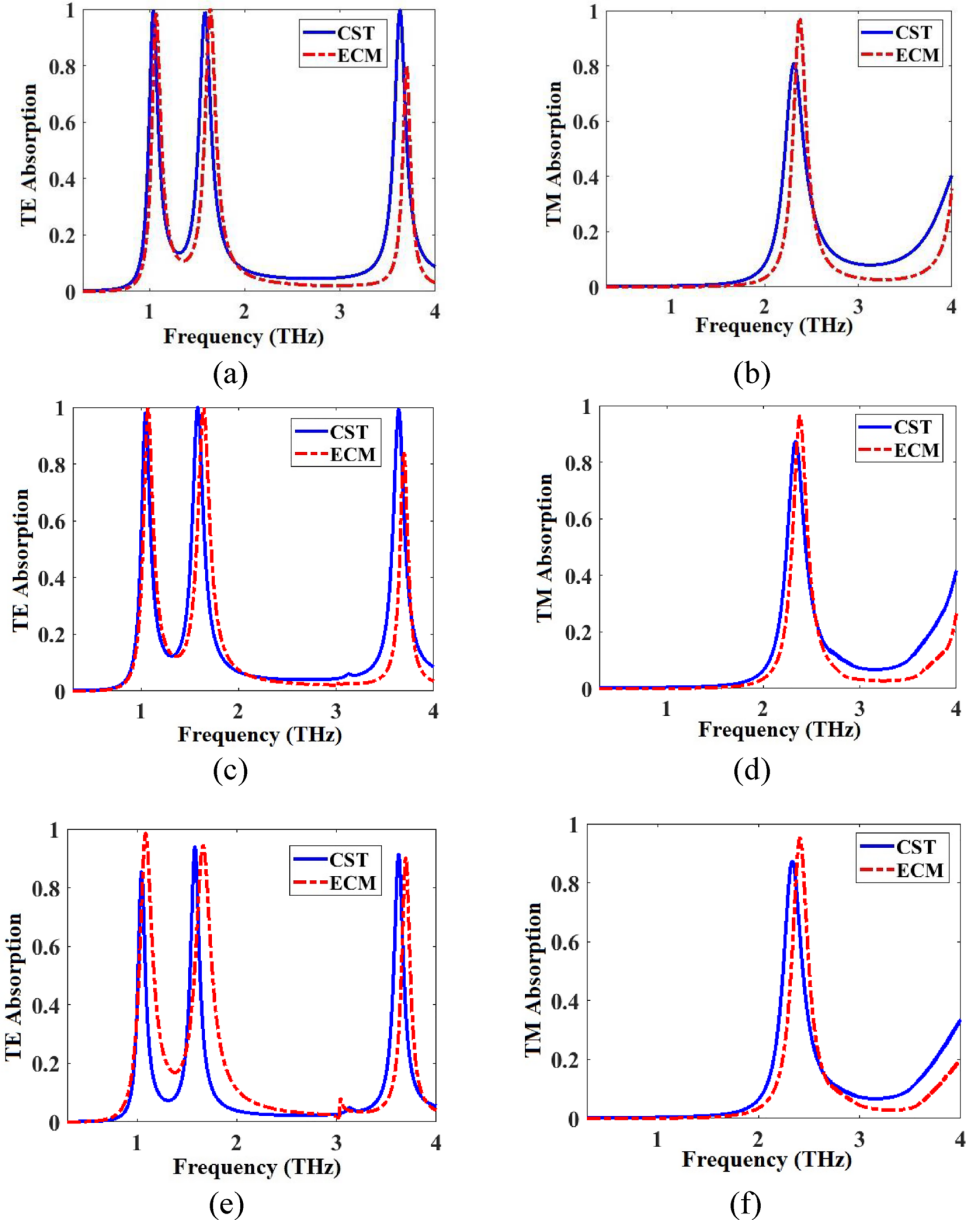
Absorption spectra of the metamaterial absorber of Fig. 1 obtained by CST and ECM in (**a**) TE mode for θ_in_ = 0, (**b**) TM mode for θ_in_ = 0, (**c**) TE mode for θ_in_ = 30, (**d**) TM mode for θ_in_ = 30, (**e**) TE mode for θ_in_ = 60, and (**f**) TM mode for θ_in_ = 60.

TE and TM absorption spectra for the metamaterial of Fig. 1 obtained by CST and ECM for three different values of θ_in_ are given in Fig. 9. Obtained spectra with both methods are in good agreement and the ECM works for any oblique incident angle of the illuminated wave θ_in_.

**Figure 10:**
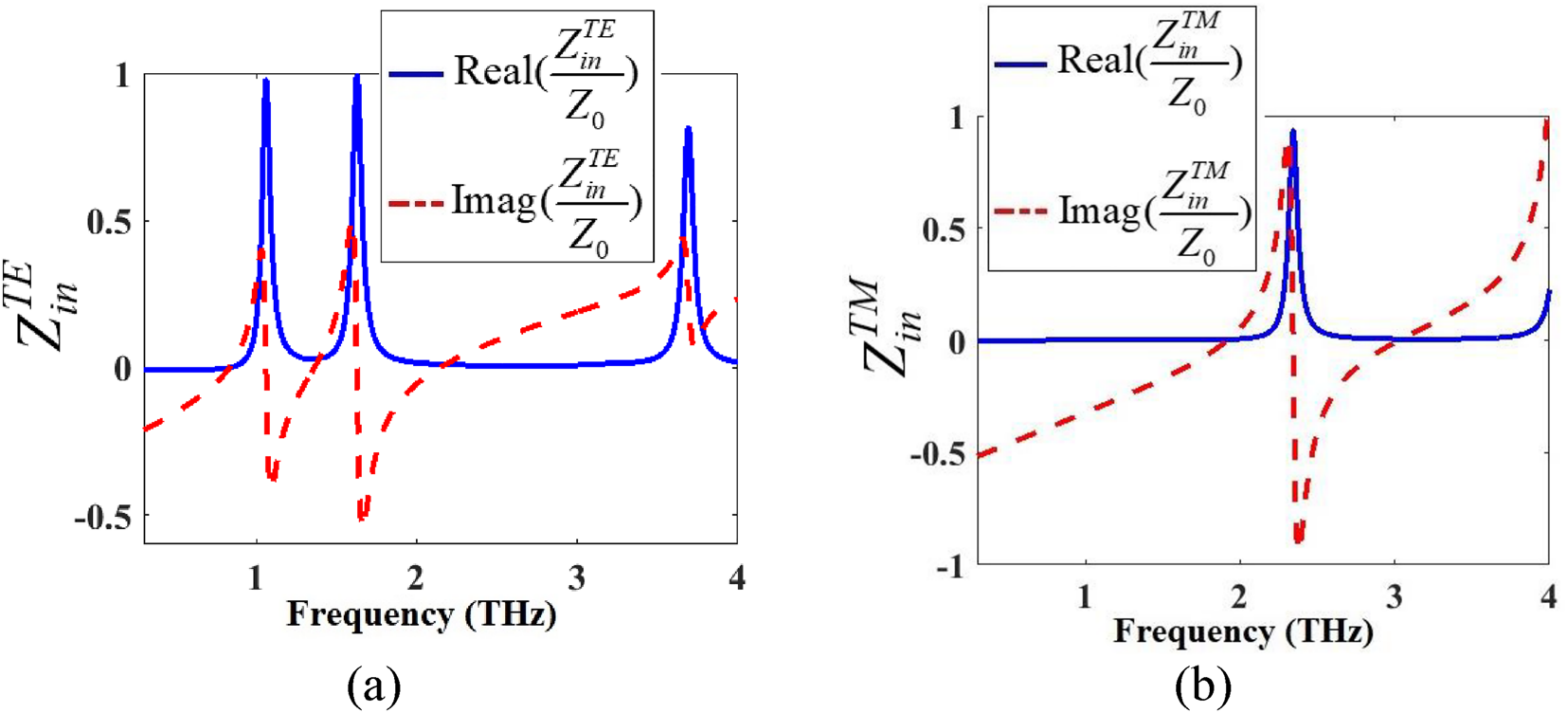
Normalized input impedance of the anisotropic metamaterial absorber of Fig. 1 in (**a**) TE and (**b**) TM modes with properties respectively presented in Fig. 9a,b.

The real and imaginary parts of the normalized input impedances in TE and TM modes (${Z}_{in}^{TE}/{Z}_{o}$ and ${Z}_{in}^{TM}/{Z}_{o}$) of the proposed anisotropic metamaterial absorber of Fig. 1 with the properties presented in Fig. 9a,b are shown in Fig. 10a,b, respectively. Z_0_ is the vacuum impedance equal to 377 Ω.

As shown in the curve obtained by ECM in Fig. 9a, absorption peaks in the first and second peaks reach 1 while the absorption in the third peak reaches 0.8. As shown in Fig. 10a, the imaginary part of the normalized input impedance is zero at the first and second absorption peaks and 0.27 at the third absorption peak. Also, the real part approaches 1 in the first and second peaks while 0.82 in the third peak. As shown in the curve obtained by ECM in Fig. 9b, absorption peak reach 1. As shown in Fig. 10b, the imaginary part of the normalized input impedance is zero at the absorption peak. Also, the real part approaches 1 in the absorption peak. These characteristics show the impedance matching condition to achieve the perfect absorption at the resonance peaks (Fig. 10).

**Figure 11:**
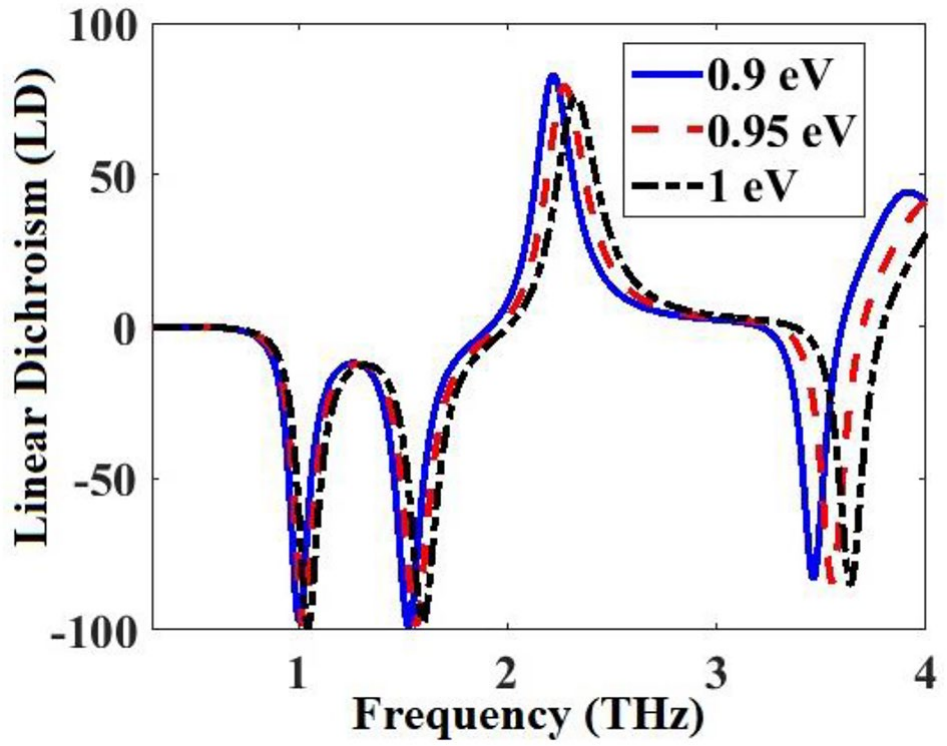
Linear dichroism (LD: the difference in absorbance for TE and TM polarized waves) of the metamaterial absorber of Fig. 1 obtained by Eq. (29) for three different values of graphene Fermi energy *E*_*f*_.

The linear dichroism (LD: the difference in absorbance for TE and TM polarized waves) vs frequency for the metamaterial of Fig. 1 obtained by Eq. (29) for three different values of graphene Fermi energy *E*_*f*_ are shown in Fig. 11. The maximum LD reaches 99% for this metamaterial. By increasing of *E*_*f*_, the resonance frequencies of the LD spectra increase, showing a blueshift. This is because the real part of the *β* decreases as the *E*_*f*_ increases (Eq. (4) in^20^). So, the resonances increase with the increase of *E*_*f*_.

The results of this work are compared with some previously published works in Table 1. The performance of the metamaterial in this work is improved compared to the metamaterial containing of two gaps (without the connecting bar) in^20^.

**Table 1:** Comparison of this work with some previously published works.

	Frequency range (THz)	Max. absorption (%)	Max. linear dichroism (LD)	Max. number of absorption bands
^5^	0.1–5.5	100	100	Two
^20^one gap structure	1–5.5	98.8	94	Three
^20^two gaps structure	1–5.5	99	93	Two
^20^three gaps structure	1–5.5	98.6	87	Three
^20^four gaps structure	1–5.5	88.4	73	Three
^39^	0.5–1.5	100	90	One
This work	0.3–4	100	99	Three

## Conclusion

In this study, we propose an Equivalent Circuit Modeling (ECM) approach utilizing impedance and transmission lines, implemented through a straightforward MATLAB code. The focus is on a terahertz (THz) graphene absorber with a graphene-based split square ring resonator array, featuring two gaps and a connecting bar. The design is subjected to numerical simulation using CST Microwave Studio Software, employing the finite element method (FEM). The results from both FEM and ECM exhibit good agreement. The absorber design is dynamically tunable, comprising a one-layer resonator array with two resonators per unit cell. The metamaterial absorber demonstrates a pronounced linear dichroism (LD) response, achieving 99% efficiency. The metamaterial has an absorption rate of 81% for one absorption band in transverse magnetic (TM) mode. Its three absorption bands in transverse electric (TE) mode have an average of 99.3% in each absorption band with absorption over 99%. This delineates a single absorption band for TM mode and three for TE mode. The proposed graphene absorber holds promise as a component in polarization-sensitive systems for controllable absorption and sensing applications.

## Data Availability

The datasets generated and/or analysed during the current study are available from the corresponding author on reasonable request.
